# Reproductive Neutrality of the A2 *β*-Casein Variant in Holstein Cows

**DOI:** 10.3390/ani16050741

**Published:** 2026-02-27

**Authors:** Lilla Sándorová, Ferenc Pajor, Péter Árpád Fehér, Miklós Gábor Szabari, Szilvia Áprily, Szilárd Bodó, Péter Póti, István Egerszegi, Ákos Bodnár, Viktor Stéger

**Affiliations:** 1 Department of Genetics and Genomics, Institute of Genetics and Biotechnology, Hungarian University of Agriculture and Life Sciences, H-2100 Gödöllő, Hungary; sandorova.lilla@uni-mate.hu (L.S.); feher.peter.arpad@uni-mate.hu (P.Á.F.); steger.viktor@uni-mate.hu (V.S.); 2 Department of Animal Technology and Animal Welfare, Institute of Animal Sciences, Hungarian University of Agriculture and Life Sciences, H-2100 Gödöllő, Hungary; poti.peter@uni-mate.hu (P.P.); egerszegi.istvan@uni-mate.hu (I.E.); bodnar.akos@uni-mate.hu (Á.B.); 3 Department of Precision Farming and Animal Biotechnology, Hungarian University of Agriculture and Life Sciences, Kaposvár Campus, H-7400 Kaposvár, Hungary; szabari.miklos.gabor@uni-mate.hu (M.G.S.); aprily.szilvia@uni-mate.hu (S.Á.); bodo.szilard@uni-mate.hu (S.B.)

**Keywords:** A1/A2 milk, biological relevance, CSN2 genotype, dairy cattle, fertility traits, Holstein cows, mixed-effects models

## Abstract

The *CSN2* gene encoding *β*-casein has gained increasing attention in dairy cattle breeding due to the global adoption of A2-oriented selection strategies. However, robust large-scale evidence assessing potential unintended effects on functional traits, particularly fertility, under intensive commercial conditions remains limited. This study evaluated whether selection for the *CSN2* A2 *β*-casein variant is associated with biologically relevant differences in fertility traits in Holstein cows. Reproductive and genomic data from 7826 lactation records of 2773 Holstein cows collected between 2022 and 2025 in a large commercial dairy herd were analyzed. Fertility indicators included days open, number of services per conception, calving interval, first-service conception rate, and pregnancy by 100 days in milk. Mixed-effects models accounting for repeated lactations and cow- and sire-level clustering were applied, and predefined equivalence margins were used to distinguish statistical non-significance from biological irrelevance. Across all evaluated fertility traits, differences among CSN2 genotypes (A1A1, A1A2, and A2A2) were consistently small, biologically negligible, and well within predefined equivalence margins. Differences in days open were within ±2 days, and effect sizes for count and binary traits were close to unity. Parity and calving year significantly influenced reproductive performance, whereas no CSN2 genotype × parity interactions were detected. These findings indicate that selection for the *CSN2* A2 *β*-casein variant does not compromise reproductive performance under intensive commercial management conditions. From a breeding and industry perspective, the results support the implementation of A2-oriented selection strategies without biologically meaningful adverse effects on fertility.

## Simple Summary

Demand for A2 milk has led to the rapid adoption of A2-oriented selection in dairy cattle breeding. This selection is based on variation in the *CSN2* gene encoding the milk protein *β*-casein. However, concerns remain that focusing on A2 milk production could unintentionally affect important functional traits such as fertility, especially in intensive commercial systems. In this study, reproductive performance was evaluated in Holstein cows with different CSN2 genotypes using a large dataset from a commercial dairy herd. Fertility traits included days open, number of services per conception, calving interval, first-service conception rate, and pregnancy by 100 days in milk. Differences among CSN2 genotypes were consistently small and not biologically meaningful. Reproductive performance was mainly influenced by parity and year, rather than CSN2 genotype. These results indicate that selection for the A2 *β*-casein variant does not compromise fertility under intensive production conditions and can be implemented without adverse effects on reproductive performance.

## 1. Introduction

Reproductive performance is a key determinant of profitability, sustainability, and animal welfare in modern dairy production systems [1,2]. Common fertility indicators such as days open, conception rate, number of services per conception, and calving interval are widely used to evaluate reproductive efficiency at both herd and population levels [3]. Despite their high economic importance, fertility traits generally exhibit low heritability and are strongly influenced by environmental, management, and physiological factors, which limits the effectiveness of direct genetic improvement [4,5,6]. Consequently, increasing attention has been directed toward evaluating whether selection targeting economically important loci may have unintended correlated effects on reproductive performance [7,8].

Among milk protein genes, *CSN2*, encoding *β*-casein, has received increasing attention due to the rapid expansion of A2-oriented breeding strategies [9,10,11]. The A2 variant differs from the A1 variant by a single amino acid substitution at position 67 [12,13]. Although differences in digestive peptide release have been discussed in relation to human health [14,15,16,17,18], causal relationships between *β*-casein variants and specific health outcomes remain debated [9,19,20]. In practice, however, breeding decisions are largely influenced by consumer demand and market differentiation, which have accelerated the global implementation of A2-oriented selection and contributed to increasing A2 allele frequencies in several Holstein populations worldwide [10,21,22].

While *β*-casein primarily affects milk protein composition and technological properties [23,24], selection targeting milk protein genes may theoretically influence reproductive performance through established physiological relationships between milk production, energy balance, endocrine regulation, and fertility during early lactation [8,25,26,27,28]. Given the well-documented antagonistic relationship between milk production and fertility in high-producing dairy cows [1,2,28], it is therefore important to verify that selection for specific milk protein variants does not result in unintended correlated responses in functional traits.

Only a limited number of studies have directly evaluated the association between β-casein genotypes and fertility traits in dairy cattle. Lu et al. [29] reported no significant effects of the A1 and A2 *β*-casein variants on reproductive traits, including days from the start of mating to conception, pregnancy rate at first service, submission rate at 21 days, and pregnancy rates at 21 and 42 days, in Holstein, Jersey, and crossbred cows under New Zealand production systems. Similarly, Arens et al. [30] reported no association between CSN2 genotype and conventional fertility traits in organic Holstein cows, although differences in survival during first lactation were detected.

Despite the rapid adoption of A2-oriented breeding strategies, large-scale evidence assessing potential reproductive consequences under intensive commercial dairy production conditions remains limited. Therefore, the objective of the present study was to evaluate whether *β*-casein genotypes (A1A1, A1A2, and A2A2) are associated with biologically relevant differences in multiple fertility traits in Holstein cows using a large commercial dataset and mixed-effects models accounting for repeated measurements and hierarchical data structure.

## 2. Materials and Methods

### 2.1. Population Description, Genomic Characterization and Fertility Traits

This observational study was conducted in a large commercial Holstein dairy herd in Hungary, in which all cows were maintained under uniform management and environmental conditions throughout the study period. Animals were housed in free-stall barns, milked three times daily in a rotary milking system, and fed a standardized total mixed ration formulated to meet or exceed the nutritional requirements of lactating Holstein cows [31]. The ration was designed to support metabolic adaptation during early lactation and to minimize negative energy balance through adequate energy density and commonly applied nutritional strategies. Reproductive management followed conventional farm practices, including visual heat detection supported by automated activity monitoring, artificial insemination performed by trained technicians, and pregnancy diagnosed by transrectal ultrasonography 30–40 days after insemination [32]. Ovulation synchronization was routinely based on an Ovsynch protocol applied at the herd level according to established farm procedures. Reproductive and nutritional management practices were uniformly applied across all cows and were not adjusted based on CSN2 genotype.

Fertility and calving records were extracted from routine herd-management databases between 2022 and 2025. Available information included calving dates, insemination dates, insemination outcomes, parity, calving year, and cow identity. Data quality screening involved the removal of clearly implausible observations (e.g., days open > 400 days), in accordance with standard recommendations for large-scale fertility analyses in dairy cattle [4,8]. To avoid bias from potentially erroneous pre-computed intervals in the management software, the calving interval was not taken from the raw interval field. Instead, calving interval was recalculated from linked consecutive calving dates for cows with ≥2 lactations; derived values were retained when both calving dates were present and internally consistent. Consequently, a small number of verified derived calving intervals exceeded 600 days and were kept in the dataset, reflecting true prolonged intervals rather than data-entry errors. Parity was initially recorded as an integer (1–8) and subsequently grouped into four categories (1, 2, 3, and ≥4) for analytical purposes.

All fertility records were linked to each animal’s CSN2 *β*-casein genotype using the herd’s internal genomic information system. All cows included in the study had previously been genotyped for *β*-casein variants by Sanger sequencing targeting exon 7 of the *CSN2* gene, as described in detail elsewhere [22,33]. Briefly, PCR amplicons covering the diagnostic SNP at codon 67 were sequenced, and chromatograms were quality-trimmed (minimum Phred score Q20) and aligned to the bovine *CSN2* reference sequence. Genotypes were assigned based on diagnostic exon-7 SNP positions, with heterozygous calls defined by clearly resolved double peaks at the target nucleotide position. Samples showing ambiguous peak patterns or insufficient sequence quality were re-amplified and re-sequenced to ensure accurate genotype determination.

The A1/A2 polymorphism represents a single nucleotide substitution resulting in a histidine-to-proline change at amino acid position 67. For herd-level functional classification, genotypes were grouped into A1-type, mixed A1/A2-type, or A2-type categories according to the amino acid present at position 67 (His67 vs Pro67), as previously described [14,33]. These functional genotype categories were used consistently in all downstream reproductive analyses.

The study population consisted of registered Holstein-Friesian dairy cows maintained under intensive commercial management. No structured crossbreeding program was implemented during the study period. Mean age at calving was 3.12 ± 1.39 years (range: 1.51–9.53 years). Age at calving was strongly correlated with parity (r = 0.98); therefore, parity class was used as the primary biological descriptor in statistical analyses.

To ensure a transparent description of the dataset structure, all lactation records were classified into four biologically meaningful parity categories (parity 1, 2, 3, and ≥4) within each CSN2 genotype. This classification reflects well-recognized physiological differences between lactations and allows for appropriate representation of population heterogeneity in subsequent statistical models. The distribution of lactation records across CSN2 genotypes and parity classes is summarized in Table 1. The study population comprised 2773 dairy cows, contributing a total of 7826 lactation records. The median number of lactations per cow was three (interquartile range: 2–4), with individual cows contributing between one and six lactations to the analysis. The dataset included offspring from 159 sires, allowing estimation of sire-level random variance in the mixed-effects models. Each lactation contributed at most one observation per fertility trait.

All procedures were part of routine farm management and did not involve any experimental interventions beyond standard practice. The study protocol, including the use of production and reproduction data, was reviewed and approved by the Institutional Animal Welfare Body of the Hungarian University of Agriculture and Life Sciences, Szent István Campus (MATE SZIC MÁB /1113-1/2024; approval issued on 10 July 2024). According to the official certification, the study falls outside the scope of EU Directive 2010/63/EU and therefore did not require an experimental animal permit.

Five reproductive indicators were evaluated:

- Days open (DO): days from calving to conception.
- Number of services per conception (NSC): total number of inseminations required to achieve pregnancy.
- Calving interval (CI): interval between two consecutive calvings (evaluated only for cows with ≥2 lactations).
- First-service conception rate (FSCR): a binary variable indicating conception at first insemination (1 = success; 0 = failure).
- Pregnancy by 100 days in milk (PR100): a binary indicator denoting pregnancy status at 100 DIM.

These indicators represent widely accepted metrics in dairy cattle reproductive monitoring and genetic evaluation and are routinely applied in large-scale fertility research [4].

### 2.2. Statistical Analysis

In studies evaluating fertility traits in dairy cattle, statistically detectable differences are not necessarily biologically or economically meaningful. Therefore, in addition to conventional hypothesis testing, the present study explicitly evaluated biological equivalence using predefined equivalence margins for each fertility trait. These margins were defined a *priori* based on established industry benchmarks and published literature regarding thresholds considered economically negligible in intensive Holstein production systems.

For interval traits, differences in days open (DO) below approximately 4–6 days are generally regarded as economically trivial at the herd level, as they correspond to only marginal changes in reproductive efficiency and replacement risk [2,4,8]. Consequently, differences smaller than ±5 days are unlikely to have a measurable economic impact under commercial conditions. Based on these considerations, a conservative equivalence margin of ±5 days was defined for days open, and ±7 days for calving interval, reflecting the greater biological and economic variability of the latter trait. Sensitivity analyses using narrower and wider equivalence thresholds, defined separately for each fertility trait, yielded identical qualitative conclusions.

For the number of services per conception (NSC), biological equivalence was defined as a rate ratio (RR) between 0.90 and 1.10, corresponding to differences smaller than approximately ±0.2 inseminations per conception, which are generally considered negligible in commercial fertility monitoring [3,4]. For binary fertility traits—first-service conception rate (FSCR) and pregnancy by 100 days in milk (PR100)—odds ratios (ORs) between 0.90 and 1.10 were considered biologically equivalent, representing changes that are unlikely to translate into economically meaningful differences in herd reproductive performance. Equivalence margins were intentionally defined independently of the observed data and prior to analysis and conservatively chosen to ensure that even small but potentially relevant differences would be detectable, rather than being derived from trait-specific economic weights. Sensitivity analyses using narrower (±3 days; OR/RR 0.95–1.05) and wider (±7 days; OR/RR 0.85–1.15) equivalence thresholds yielded identical qualitative conclusions, with all CSN2 genotype contrasts remaining biologically negligible.

All statistical analyses were performed using R software (version 4.3.2; R Core Team, 2023) [34]. Because individual cows contributed repeated observations across lactations and the data were hierarchically structured, all fertility traits were analyzed using mixed-effects models to account for non-independence among observations and clustering within cows and sires [35,36].

Across all models, CSN2 genotype (A1A1, A1A2, A2A2), parity class (1, 2, 3, ≥4), and calving year were included as fixed effects. Random intercepts for cow and sire were considered where appropriate and retained only when they improved model convergence and explained a non-negligible proportion of variance, as assessed by variance component estimates and overall model stability. Model selection decisions regarding random effects were based on variance component estimates and model stability rather than formal hypothesis testing.

Calving season was evaluated as a categorical fixed effect in preliminary models but was excluded from final analyses because it did not improve model fit, as assessed by Akaike’s Information Criterion (AIC), nor did it materially alter CSN2 genotype effect estimates. Days open (DO) and calving interval (CI) exhibited right-skewed distributions and were therefore analyzed after natural logarithmic transformation of the response variable:

$$y_{i}^{*}=ln(y_{i})$$

where $y_{i}^{*}$ denotes the observed value of days open (DO) or calving interval (CI) for the ith observation. Log-transformed DO and CI were analyzed using linear mixed-effects models of the form:

$$y_{ij}^{*}=\beta_{0}+\beta_{1}{\mathrm{Genotype}}_{i}+\beta_{2}{\mathrm{Parity}}_{i}+\beta_{3}{\mathrm{Year}}_{i}+u_{\mathrm{cow}(j)}+u_{\mathrm{sire}(j)}+\varepsilon_{ij}$$

where $u_{\mathrm{cow}(j)}$ and $u_{\mathrm{sire}(j)}$ denote random intercepts for cow and sire, respectively, and $\varepsilon_{ij}$ is the residual error term. For calving interval, only the random cow intercept was retained based on variance contribution and model stability. Models were fitted using the lmer function in the lme4 package [36]. Adjusted marginal means were back-transformed to the original scale for ease of interpretation.

The number of services per conception (NSC) is a count variable and exhibited marked overdispersion relative to the Poisson assumption, as indicated by residual diagnostics and dispersion statistics. NSC was therefore analyzed using a negative binomial mixed-effects model fitted with the glmmTMB package (version 1.1.13) [37] following recommendations for overdispersed count data [38]. The linear predictor was specified as:

$$log(\mu_{ij})=\beta_{0}+\beta_{1}{\mathrm{Genotype}}_{i}+\beta_{2}{\mathrm{Parity}}_{i}+\beta_{3}{\mathrm{Year}}_{i}+u_{\mathrm{cow}(j)}$$

where $\mu_{ij}$ denotes the expected number of inseminations for the ith observation. Exponentiated fixed-effect contrasts from the count model were interpreted as rate ratios (RRs).

Binary fertility traits—first-service conception rate (FSCR) and pregnancy by 100 days in milk (PR100)—were analyzed using logistic mixed-effects models fitted with the glmer function in lme4 [36]. The general model form was:

$$\mathrm{logit}(p_{ij})=\beta_{0}+\beta_{1}{\mathrm{Genotype}}_{i}+\beta_{2}{\mathrm{Parity}}_{i}+\beta_{3}{\mathrm{Year}}_{i}+u_{\mathrm{cow}(j)}+u_{\mathrm{sire}(j)}$$

where $p_{ij}$ denotes the probability of a positive reproductive outcome. For FSCR, only the random cow intercept was retained, whereas for PR100, both cow and sire random intercepts were included based on their contribution to explained variance and model stability. Exponentiated fixed-effect contrasts from the logistic models were interpreted as odds ratios (ORs). Odds ratios are reported for binary outcomes as they arise naturally from the logistic mixed-effects framework; given the moderate outcome prevalence in the present dataset, odds ratios closely approximate relative risks.

Adjusted marginal means and pairwise contrasts were computed using the emmeans package (version 2.0.0) [39], with Tukey adjustment applied to all genotype comparisons to control for multiple testing. CSN2 genotype × parity interactions were evaluated for all fertility traits and were retained in final models only when statistically significant and biologically interpretable.

Model diagnostics were performed using the DHARMa package (version 0.4.7) [40], including simulation-based assessments of residual uniformity, dispersion, and potential outliers. Collinearity among fixed effects was assessed using variance inflation factors, with all values < 2 indicating the absence of problematic multicollinearity. For all mixed models, marginal (*R*^2^*ₘ*) and conditional (*R*^2^*c*) coefficients of determination were calculated following Nakagawa and Schielzeth [41] and Nakagawa et al. [42].

Statistical significance was assessed at *p* < 0.05 for all analyses. Interpretation of results emphasized effect size magnitude and biological relevance in addition to statistical significance. The interpretation of results focused on effect sizes and predefined equivalence margins, reflecting the growing consensus that statistical non-significance alone is insufficient to assess biological or practical relevance in applied animal and livestock research [43,44,45].

## 3. Results

### 3.1. Descriptive Statistics of Fertility Traits

Descriptive statistics are presented to characterize data distribution, whereas all inferential analyses are based on mixed-effects models. After data screening, a total of 7826 lactation records from 2773 Holstein cows were retained for analysis. The distribution of CSN2 genotypes across lactations was A1A1 (*n* = 730), A1A2 (*n* = 3599) and A2A2 (*n* = 3497). Descriptive statistics of the main fertility traits are presented in Table 2. Adjusted marginal means are presented on the original scale after back-transformation from log-models and therefore differ from unadjusted descriptive means. Adjusted means reflect model-based estimates accounting for parity and year and are therefore not expected to equal raw averages.

Mean days open (DO) was 73.6 days (median 76; range 11–303), the number of services per conception (NSC) averaged 1.97 (median 2; range 1–9), and mean calving interval (CI) was 382.5 days (median 371; range 312–659). Clear differences in fertility traits were observed across parity classes, with higher-parity cows showing longer DO and CI values and requiring more inseminations. Significant year-to-year variation was also observed for all fertility traits, indicating appreciable environmental variability despite uniform herd management.

Records with missing genotype or fertility information were excluded prior to analysis. The large sample size ensured high statistical power to detect even small genotype-related differences in fertility traits, had such differences been present.

### 3.2. Effect of CSN2 Genotype on Days Open (DO)

**Figure 1:**
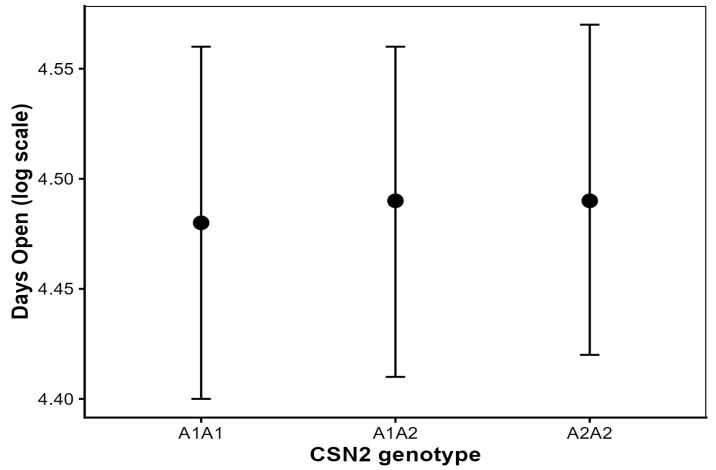
Adjusted means (±95% confidence intervals) for days open by CSN2 genotype in Holstein cows. Model estimates are presented as back-transformed marginal means with 95% confidence intervals.

The CSN2 genotype was not significantly associated with days open. Estimated differences between CSN2 genotypes were small (≤2 days) and were considered biologically negligible. On the original scale (back-transformed marginal means), genotype contrasts corresponded to absolute differences ≤2 days. Adjusted marginal means were nearly identical across A1A1, A1A2 and A2A2 cows, corresponding to approximately 88–89 days after back-transformation (Figure 1). Model-based adjusted means are higher due to parity and year adjustment. Pairwise comparisons confirmed the absence of statistically significant differences among genotypes (all Tukey-adjusted *p* > 0.60).

Parity had a strong effect on days open (*p* < 0.001), with progressively longer intervals observed from first to higher parities, while calving year showed a diminished but significant effect (*p* < 0.01). No significant CSN2 genotype × parity interaction was detected (*p* > 0.10), indicating a consistent lack of genotype effect across lactations. Random cow effects accounted for approximately 20–22% of total phenotypic variance, whereas sire effects were small.

### 3.3. Effect of CSN2 Genotype on Number of Services per Conception (NSC)

**Figure 2:**
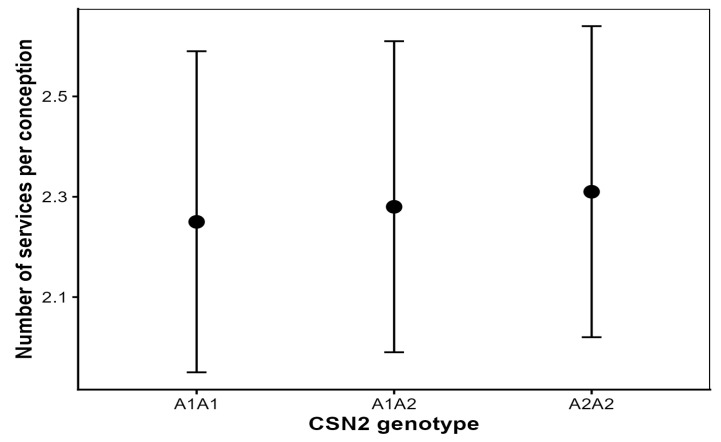
Adjusted means (±95% confidence intervals) for number of services per conception (NSC) by CSN2 genotype in Holstein cows.

The CSN2 genotype was not associated with the number of services per conception. Adjusted marginal means were 2.25, 2.28 and 2.31 services per conception for A1A1, A1A2 and A2A2 cows, respectively (Figure 2), and none of the pairwise contrasts between genotypes were statistically significant (all Tukey-adjusted *p* > 0.60).

Parity had a strong effect on NSC (*p* < 0.001), with higher-parity cows requiring more inseminations, and calving year also showed a significant effect (*p* < 0.001). No significant CSN2 genotype × parity interaction was detected (*p* > 0.10), indicating a consistent lack of genotype effect across parities.

Random cow effects accounted for approximately 18% of the total variance, indicating substantial individual-level heterogeneity in insemination outcomes.

Effect size estimates were extremely small: the rate ratio comparing A2A2 with A1A1 cows was approximately 1.02, indicating differences far below thresholds considered biologically relevant for NSC.

### 3.4. Effect of CSN2 Genotype on Calving Interval (CI)

**Figure 3:**
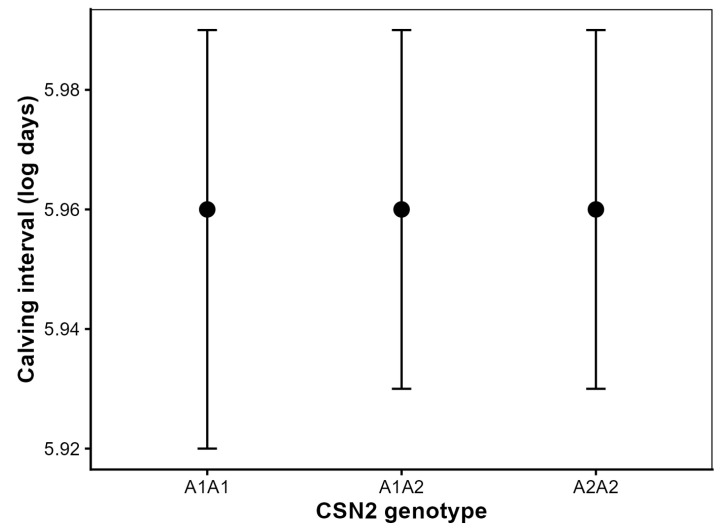
Adjusted means (±95% confidence intervals) for calving interval (CI) by CSN2 genotype in Holstein cows.

Calving interval was not associated with CSN2 genotype in cows with at least two consecutive calvings (*n* = 3115). Back-transformed marginal mean differences between genotypes were <5 days and thus of limited biological relevance. Adjusted marginal means were identical across A1A1, A1A2 and A2A2 genotypes, corresponding to approximately 388 days after back-transformation (Figure 3). Pairwise comparisons confirmed the absence of statistically significant differences among genotypes (all Tukey-adjusted *p* > 0.80).

Parity had a significant effect on calving interval (*p* < 0.001), with progressively longer intervals observed in higher-parity cows, and calving year also showed a significant effect (*p* < 0.001). No significant CSN2 genotype × parity interaction was detected (*p* > 0.10), indicating a consistent lack of genotype effect across parities.

Random cow effects explained a substantial proportion of total phenotypic variance (approximately 24%), whereas fixed effects accounted for only a small share of total variation (*R*^2^*m* = 0.024).

### 3.5. Effect of CSN2 Genotype on First-Service Conception Rate (FSCR)

First-service conception rate was not associated with *CSN2* genotype. Adjusted probabilities of conception at first insemination were 0.203, 0.214, and 0.198 for A1A1, A1A2, and A2A2 cows, respectively, and none of the pairwise genotype contrasts were statistically significant (all Tukey-adjusted *p* > 0.50). Corresponding odds ratios were close to unity (0.94–1.06), indicating no biologically meaningful differences in first-service conception outcomes among CSN2 genotypes.

Parity had a significant effect on FSCR (*p* = 0.007), with lower conception probabilities observed in higher-parity cows, and calving year also significantly influenced FSCR (*p* < 0.001). No significant CSN2 genotype × parity interaction was detected (*p* > 0.10), indicating a consistent lack of genotype effect across parities. Random cow effects accounted for a substantial proportion of total variability in FSCR, whereas sire effects were comparatively small. Model diagnostics indicated adequate fit, with no evidence of influential observations or violations of model assumptions.

### 3.6. Effect of CSN2 Genotype on Pregnancy by 100 Days in Milk (PR100)

Pregnancy by 100 days in milk was not associated with the CSN2 genotype. Adjusted probabilities of being pregnant by 100 days in milk were 0.482, 0.484, and 0.468 for A1A1, A1A2, and A2A2 cows, respectively, and none of the pairwise genotype contrasts were statistically significant (all Tukey-adjusted *p* > 0.58). Corresponding odds ratios were close to unity (0.94–1.06), and all effect estimates fell well within predefined biological equivalence margins, indicating no biologically meaningful differences in pregnancy by 100 days in milk among CSN2 genotypes.

Parity had a significant effect on PR100 (*p* < 0.001), with lower pregnancy probabilities observed in higher-parity cows, and calving year also significantly influenced PR100 (*p* < 0.001). No significant CSN2 genotype × parity interaction was detected (*p* > 0.10), indicating a consistent lack of genotype effect across parities. Random cow and sire effects accounted for a substantial proportion of total variability in PR100. Model diagnostics indicated adequate fit, with no evidence of influential observations or violations of model assumptions.

### 3.7. Correlations Among Fertility Traits

**Figure 4:**
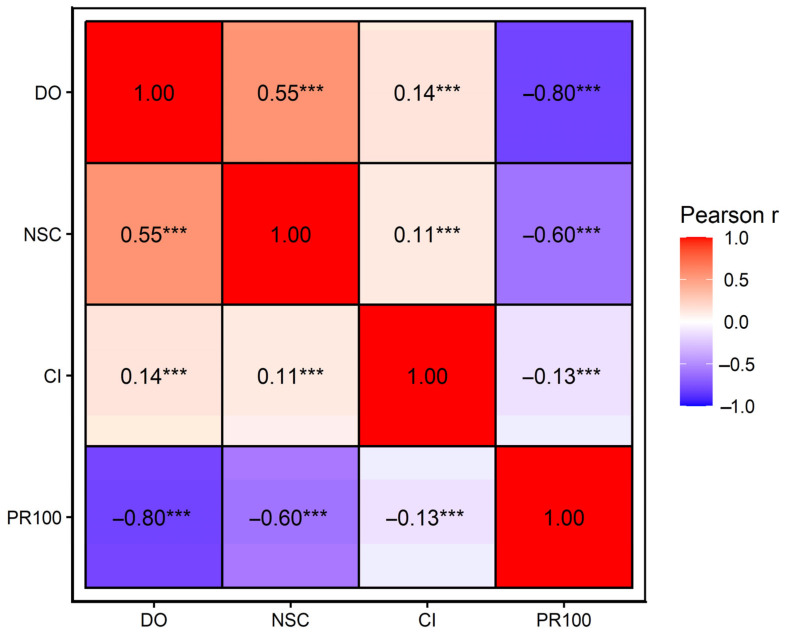
Pearson correlation heatmap among fertility traits in Holstein cows; cell values show Pearson’s r, colors indicate the direction and magnitude of correlations, and asterisks denote significance (*** *p* < 0.001). Notes: DO: Days open; NSC: Number of services per conception; CI: Calving interval; PR100: Pregnancy by 100 days in milk. Values represent Pearson correlation coefficients (*n =* 3115).

Pearson correlation analysis (*n =* 3115) revealed strong associations among several fertility traits. Correlation analyses were restricted to cows with complete data for all traits, including calving interval. Correlation analysis was included to contextualize the interrelationships among fertility traits rather than to infer causality. Because PR100 is a thresholded indicator of conception by 100 days in milk, its correlation with days open (DO) partly reflects this shared definition and should be interpreted descriptively. This strong association is therefore expected and arises from the definitional overlap between PR100 and DO, rather than indicating redundancy or analytical circularity. Days open (DO) was positively correlated with the number of services per conception (NSC) (r = 0.55) and negatively correlated with pregnancy by 100 days in milk (PR100) (r = −0.80). NSC was also negatively correlated with PR100 (r = −0.60). Correlations involving calving interval (CI) were weaker (|r| ≤ 0.14). All correlations were statistically significant (*p* < 0.001). The overall correlation structure among fertility traits is illustrated in Figure 4.

These strong interrelationships indicate that fertility traits are tightly connected, suggesting that the observed lack of CSN2 genotype effects is unlikely to be explained solely by compensatory patterns among the evaluated fertility indicators. Across all fertility traits, estimated CSN2 genotype effects were consistently small and entirely within predefined biological equivalence margins, providing evidence against biologically meaningful differences in reproductive performance.

### 3.8. Model Explanatory Performance

Model performance was assessed based on variance components, the relative contributions of fixed and random effects, and residual diagnostics. Across all fertility traits, the fixed effects included in the models (parity and calving year) explained only a modest proportion of total phenotypic variation.

For days open (DO) and calving interval (CI), a substantial share of explained variation was attributed to the random cow intercept, indicating marked between-cow heterogeneity beyond the measured covariates. In contrast, models for number of services per conception (NSC) and first-service conception rate (FSCR) showed lower overall explanatory power, while the pregnancy by 100 days in milk (PR100) model showed a somewhat greater contribution of fixed effects, alongside notable random variation at both cow and sire levels.

**Figure 5:**
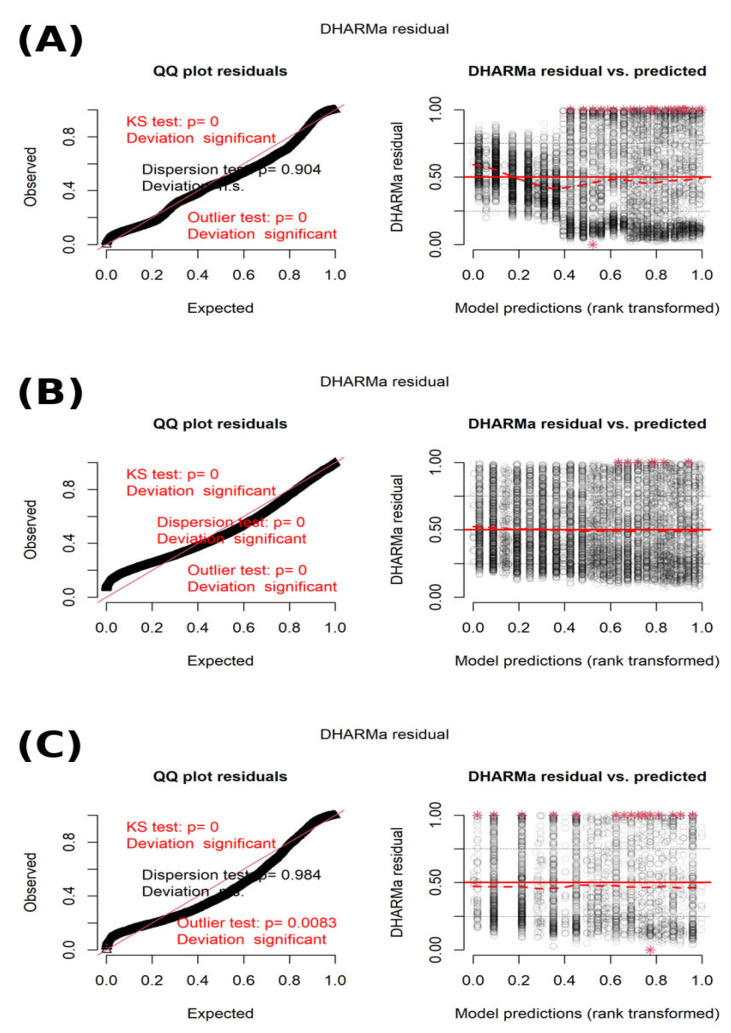
DHARMa residual diagnostics for mixed-effects models of fertility traits. Simulation-based residual diagnostics for mixed-effects models of (**A**) days open (DO), (**B**) number of services per conception (NSC), and (**C**) calving interval (CI), assessed using the DHARMa package. Formal goodness-of-fit tests shown in red are known to be highly sensitive in large datasets and may indicate statistically significant but biologically negligible deviations from idealized assumptions. Interpretation therefore focuses on visual assessment of residual uniformity, dispersion, and residual–prediction relationships, which did not reveal systematic patterns or violations that would materially affect model inference. Red text indicates statistically significant results of DHARMa diagnostic tests (*p* < 0.05). Red asterisks (*) denote observations flagged as potential outliers by the DHARMa outlier test.

Across all models, the CSN2 genotype contributed negligibly to explained variance, consistent with the absence of significant genotype effects observed in the trait-specific analyses. Model diagnostics based on simulation-derived residuals indicated adequate model fit, with no evidence of systematic residual patterns affecting inference (Figure 5A–C).

## 4. Discussion

Using a large commercial dataset and mixed-effects models accounting for repeated measurements and hierarchical data structure, the present study evaluated whether selection for the *CSN2* A2 *β*-casein variant is associated with biologically meaningful differences in reproductive performance in Holstein cows. Across all evaluated fertility traits—days open, number of services per conception, calving interval, first-service conception rate, and pregnancy by 100 days in milk—estimated genotype differences were small and fell within predefined biological equivalence margins. The absence of detectable genotype effects was consistent across parity classes and calving years, indicating no evidence of context-dependent or parity-specific influences under the studied commercial management conditions. This pattern is consistent with the well-established low heritability and strong environmental sensitivity of fertility traits in dairy cattle [4,5,6,28].

A key contribution of the present study lies in the explicit distinction between statistical non-significance and biological irrelevance. With more than 7800 lactation records, the analytical framework provided sufficient statistical power to detect modest genotype-related differences. Effect estimates were consistently close to unity (for count and binary traits) or within narrow absolute ranges (for interval traits), remaining well inside predefined equivalence thresholds. These findings support the interpretation that A2-oriented selection is reproductively neutral with respect to the fertility traits evaluated. Such an approach aligns with the increasing emphasis on effect size interpretation and biological relevance in animal breeding research, where statistical significance alone does not necessarily imply economic or biological importance [43,44,45].

The A1/A2 difference represents a well-characterized single nucleotide polymorphism within the *CSN2* gene and reflects established standing genetic variation in bovine populations rather than an ongoing mutation process [10,12,13]. While mutation–fitness trade-offs have been discussed in broader evolutionary contexts [46,47], a comparable mechanism linking the CSN2 A1/A2 polymorphism to reproductive performance in dairy cattle has not been demonstrated. Although *β*-casein variants are known to influence milk protein composition and technological properties [10,23,24], current evidence does not indicate a direct physiological pathway linking *β*-casein polymorphism to female reproductive function in dairy cattle [19]. Reviews addressing milk protein genetics and dairy cattle fertility describe these biological domains as largely independent [8,10]. If any association were to exist, it would most plausibly arise indirectly through relationships between milk production, energy balance, metabolic adaptation, and endocrine regulation during early lactation [8,25,26,27,28]. In this context, the present results further indicate that the CSN2 genotype is unlikely to represent a major source of reproductive variation under intensive commercial conditions.

It is important to distinguish between genotype and gene expression when interpreting these findings. The present study evaluated associations between CSN2 genotype—a stable DNA variant—and fertility outcomes; genotype itself is not modified by environmental conditions. In contrast, reproductive performance is strongly influenced by management, nutrition, and physiological status [2,4,28,48,49]. To reduce environmental heterogeneity, the analysis controlled for parity and calving year and was conducted within a uniform herd environment. While genotype × environment interactions cannot be entirely excluded, the absence of genotype effects across parity classes and years suggests that such interactions, if present, are unlikely to be of practical relevance under comparable intensive management systems. No significant genotype × parity interactions were detected, further supporting the absence of context-dependent genotype effects under the studied conditions.

The results are consistent with previous investigations examining associations between *β*-casein genotypes and reproductive performance. Lu et al. [29] reported no significant effects of A1 and A2 variants on fertility traits under New Zealand production conditions, and Arens et al. [30] found no association between CSN2 genotype and conventional fertility traits in organic Holstein cows. Together with evidence that milk protein loci such as *CSN2*, *CSN3*, and *BLG* primarily influence milk composition rather than functional traits [24], these findings indicate that *β*-casein genotype is not a major determinant of fertility variation in dairy cattle.

From a breeding perspective, these findings are particularly relevant given the widespread adoption of A2-oriented selection strategies driven by market demand [10,22]. Because antagonistic relationships between milk production and fertility are well documented [1,8,25,28], concerns have been raised that selection targeting milk protein variants could indirectly compromise reproductive performance through metabolic pathways [26]. The present results suggest that, at least for the fertility traits evaluated, such unintended correlated responses are unlikely. This supports the inclusion of A2-oriented selection within balanced breeding objectives without necessitating compensatory selection pressure on fertility traits [50].

Several limitations should be acknowledged. Data were derived from a single large commercial herd operating under intensive Hungarian production conditions, including total mixed ration feeding, synchronized reproductive management, and high milk yield. Therefore, extrapolation to pasture-based, organic, or extensively managed systems—or to regions with markedly different climatic conditions or genetic backgrounds—should be made cautiously. At the same time, the single-herd design represents a methodological strength, as uniform management and environmental conditions reduce confounding and enhance internal validity when evaluating genotype–phenotype associations. Future multi-herd studies across diverse production systems would further strengthen conclusions regarding the external generalizability of these findings.

While this study focused on conventional fertility traits, it did not assess potential associations with specific health disorders or long-term survival beyond those indirectly reflected in reproductive performance. Future research integrating fertility, health, and longevity traits across multiple herds would provide a more comprehensive evaluation of the broader functional implications of CSN2 selection.

Taken together, the available evidence does not indicate that A2-oriented selection compromises fertility under intensive commercial dairy production. In the context of expanding A2-focused breeding strategies, these findings provide relevant reassurance for breeding programs and industry decision-making. Validation across multiple herds and management systems would further strengthen the generalizability of these conclusions.

## 5. Conclusions

This large-scale commercial herd study indicates that selection for the *CSN2* A2 β-casein variant does not compromise reproductive performance in Holstein cows under intensive management conditions. Genotype-related differences were small and biologically negligible across all evaluated fertility traits. Given the growing adoption of A2-based breeding strategies, these findings provide relevant reassurance for breeding and industry stakeholders. The results support the integration of A2-oriented selection into balanced dairy breeding programs without evidence of adverse effects on fertility. While confirmation across multiple herds would be valuable, the present study offers a practically relevant assessment under commercial intensive conditions.

## Figures and Tables

**Table 1:** Distribution of lactation records by CSN2 genotype and parity class (descriptive statistics only).

CSN2 Genotype	Parity 1	Parity 2	Parity 3	Parity ≥ 4
A1A1	164	173	118	275
A1A2	1308	1085	678	528
A2A2	1565	989	501	442

**Table 2:** Descriptive statistics of the main fertility traits in the Holstein herd. CI was calculated only for cows with at least two consecutive calvings.

Trait	N	Mean	Median	Min	Max
Days open (DO), days	7826	73.6	76	11	303
Number of services per conception (NSC)	7826	1.97	2	1	9
Calving interval (CI), days	3115	382.5	371	312	659

## Data Availability

Annotated R scripts used for statistical modeling are available from the corresponding author upon reasonable request.
